# A Case of mild idiopathic adulthood ductopenia and brief review of literature

**DOI:** 10.1093/gastro/gou048

**Published:** 2014-07-16

**Authors:** Aung Kaung, Vinay Sundaram, Deepti Dhall, Tram T. Tran

**Affiliations:** ^1^Department of Medicine, Cedars-Sinai Medical Center, Los Angeles, CA, USA, ^2^Center for Liver Disease and Transplantation, Cedars-Sinai Medical Center, Los Angeles, CA, USA and ^3^Department of Pathology, Cedars-Sinai Medical Center, Los Angeles, CA, USA

**Keywords:** idiopathic adulthood ductopenia, idiopathic cholestasis, primary biliary cirrhosis, small-duct primary sclerosing cholangitis

## Abstract

Mild idiopathic adulthood ductopenia (IAD) is a rare cholestatic disease of unknown cause and characterized by interlobular bile duct loss in less than 50% of the portal tracts. We describe the case of a middLe-aged male who presented with persistent elevation of transaminases and alkaline phosphatase. He had a normal biliary tree on endoscopic retrograde cholangiopathy and negative anti-mitochondrial antibody. His liver biopsy specimen showed chronic biliary disease, duct loss in 4 out of 15 portal tracts and prominent cholestasis. Based on the work-up, he likely had mild IAD. Liver transplantation would be necessary if his disease becomes progressive.

## INTRODUCTION

Idiopathic adulthood ductopenia (IAD) is a chronic cholestatic disease characterized by loss of interlobular bile ducts [1, 2]. Few cases of IAD have been reported since its first description in 1988 [1]; however, its pathogenesis remains unknown. IAD can be generally categorized into mild and severe. Mild IAD is associated with a loss of ≤50% of the interlobular bile ducts and a benign clinical course, while severe IAD is characterized by a loss of >50% of the bile ducts and a more aggressive clinical course [3–5]. A diagnosis of IAD requires exclusion of other etiologies of chronic cholestasis [1–3]. Here we describe a case of mild idiopathic adulthood ductopenia, accompanied by a brief review of the literature.

## CASE PRESENTATION

**Figure 1:**
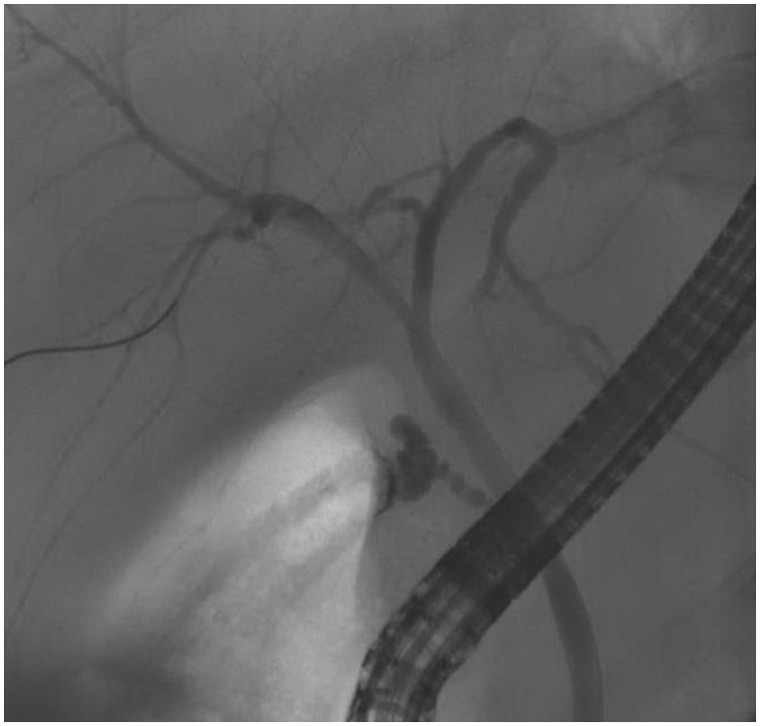
ERCP demonstrates a normal common bile duct, common hepatic duct and right and left hepatic ducts without evidence of intraluminal filling defects.

**Figure 2:**
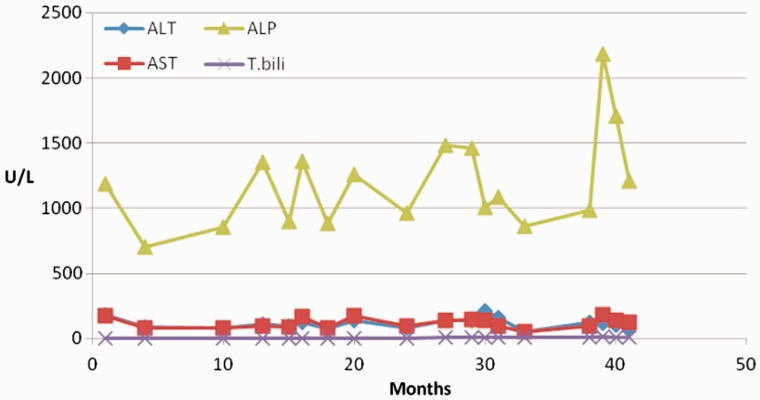
The graph shows abnormal liver enzymes despite treatment with UDCA over 3-year follow-up.

**Figure 3:**
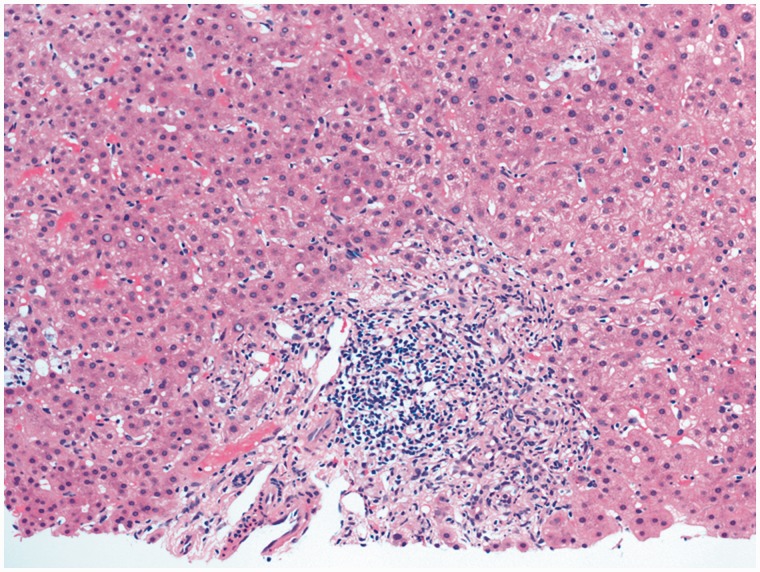
A comparison of the histological findings of the liver biopsy specimen (H&E, ×200). There is a significant number of lymphocytes in the portal tract. Interlobular bile ducts are absent.

A 50-year-old Caucasian male was referred to our clinic in late 2010 for elevated liver enzymes. About two years prior to his initial clinic visit, he reportedLy had an episode of jaundice after taking terbinafine, but it had resolved spontaneously after three months. He denied any recent use of prescription medication, herbal medication, alcohol or illicit drugs. There was no family history of recurrent cholestatic disorder or liver disease. Physical examination was significant for marked jaundice and scleral icterus but there was neither sign nor stigmata of chronic liver disease. Laboratory tests showed white blood count 3300/μL, hemoglobin 13 g/dL, platelets 186 000/μL, alanine aminotransferase (ALT) 91 U/L, aspartate aminotransferase (AST) 96 U/L, alkaline phosphatase (ALP) 712 U/L, gamma-glutamyl transpeptidase (GGT) 643 U/L, total bilirubin 2.4 mg/dL, albumin 4.3 g/dL, total protein 7.7 g/dL, international normalized ratio 1 and creatinine 1.1. Hepatitis A, B and C serology was negative. Iron studies, ceruloplasmin, alpha 1-anitrypsin, serum immunoglobulins and protein electrophoresis were normal. Anti-nuclear (ANA), anti-smooth muscle, anti-mitochondrial (AMA) and anti-liver-kidney microsome antibodies were negative. Abdominal ultrasound showed no evidence of intra- or extrahepatic biliary dilation or cholelithiasis. Magnetic resonance imaging/magnetic resonance cholangiopancreatography (MRI/MRCP) revealed normal pancreatic duct, common hepatic duct, common bile duct without ductal dilation and normal liver. Esophagogastroduodenoscopy and colonoscopy did not show evidence of inflammatory bowel disease. Further work-up with endoscopic retrograde cholangiopancreatography (ERCP) revealed normal pancreatic and biliary system, without evidence of any large- or small-duct disease (Figure 1). Ursodeoxycholic acid (UDCA) 600 mg daily was administered for his symptomatic cholestasis. In six-month and one-year follow-up visits, there was no improvement in his liver enzymes (Figure 2); however, his liver synthetic function remained intact and there was no evidence of hepatic decompensation. Given persistent elevation of liver enzymes, percutaneous liver biopsy was performed in 2013. This demonstrated chronic biliary disease with duct injury, duct loss in at least 4 out of 15 portal tracts, prominent cholestasis and mild periportal fibrosis (Figure 3). Work-up for sarcoidosis showed normal angiotensin converting enzyme (ACE) level, while mediastinal lymph node biopsy was unrevealing.

Three months later, the patient was admitted to the hospital for worsening jaundice and pruritus. His liver enzymes were elevated to ALT 167 U/L, AST 221 U/L, ALP 1726 U/L and total bilirubin 14.9 mg/dL. MRCP revealed under-distended, poorly visualized intrahepatic ducts and non-dilated common bile duct. Subsequent ERCP with biliary sphincterotomy demonstrated normal cholangiogram with minimal narrowing of the intrahepatic ducts, without evidence of changes. On discharge, his liver enzymes improved to ALT 39 U/L, AST 40 U/L, ALP 637 U/L, total bilirubin 10.8 mg/dL, and UDCA was increased to 1200 mg daily. In subsequent clinic visits, his liver enzymes were found to be persistently elevated to ALP 1500–2000 U/L and total bilirubin 20–30 mg/dL, without any clinical signs of hepatic decompensation (Figure 2).

Leading differential diagnoses were small biliary duct etiologies including AMA-negative primary biliary cirrhosis (PBC), small duct primary sclerosing cholangitis (PSC) and idiopathic adulthood ductopenia (IAD). However, the patient's gender was unusual for PBC, while extreme elevation of bilirubin and ALP without concomitant liver failure was not consistent with PSC and PBC. In addition, he did not respond to empirical treatment for autoimmune cholangiopathy, and had a negative sarcoidosis work-up. Furthermore, his age of onset and benign family medical history made hereditary disorders, such as benign recurrent intrahepatic cholestasis or progressive familial intrahepatic cholestasis, less likely. Therefore, diagnosis of IAD was made.

## DISCUSSION

Idiopathic adulthood ductopenia is a chronic cholestatic disease of unknown etiology first described by Ludwig *et al.* in 1988 [1]. In a retrospective review of 2082 cases of small-duct biliary diseases, diagnosed between 1988 and 1994, IAD accounts for only 1.2%, while primary sclerosing cholangitis and primary biliary cirrhosis constitute 93% [2].

Several etiologies have been suggested for IAD, including late-onset non-syndromic paucity of intrahepatic bile ducts, small duct primary sclerosing cholangitis without large duct involvement and without evidence of inflammatory bowel disease, non-suppurative viral cholangitis, autoimmune cholangitis or cholangitis in autoimmune hepatitis in the absence of the typical autoantibodies, genetic factors and hepatitis C [2, 6–8]. Although no familial clustering of mild IAD has been found [9], the possibility of an immunogenetic basis for disease susceptibility in patients with mild IAD has also been suggested [10].

Nonetheless, IAD remains a diagnosis of exclusion in young-to-middle-aged adults without history of infantile cholangiopathy, and is characterized by biochemical evidence of cholestatic liver disease, as well as histological evidence of ductopenia, the loss of interlobular and septal bile ducts in at least 50% of the portal tracts [1, 2]. Clinically, patients with IAD may present with episodic jaundice and pruritus or without symptoms [11]. The course of IAD can be variable and two types of IAD, associated with different prognoses, are currently recognized. Patients with Type 1 IAD have less than 50% loss of biliary ducts on liver biopsy specimens, have a more benign clinical course and better prognosis than those with more severe duct loss. Patients with Type 2 IAD have more extensive ductopenia, decompensated biliary cirrhosis and require liver transplantation earlier [3, 4].

Since IAD is associated with variable etiologies, its treatment options may vary. In patients with non-progressive IAD, UDCA has been shown to improve liver enzymes [6, 11, 12]. On the other hand, patients with progressive diseases, and those with advanced cases, will ultimately require orthotopic liver transplantation [2–4, 6, 13].

Although the diagnosis for his chronic cholestatic liver disease may not be definitive, our patient probably had Type 1 IAD, given ductopenia that affected less than 50% of the portal tracts. His clinical course also had been stable, without any hepatic decompensation, despite significantly elevated bilirubin and ALP. In the case of Type 1 IAD, it is possible that his ductopenia was contributed by various entities including small-duct PSC, AMA-negative PBC and autoimmune cholangitis. On the other hand, his chronic cholestasis could have been a manifestation of the overlap syndrome of the aforementioned biliary duct etiologies. Nonetheless, his liver enzymes had not improved with UDCA and his prognosis was unclear.

In summary, etiological factors for IAD appear to be heterogeneous and uncertain. Therefore, its clinical course and prognosis are variable and treatment options remain unclear. UDCA may not improve abnormal liver enzymes nor clinical symptoms. IAD should remain a diagnosis of exclusion in approaching patients with chronic cholestasis.

*Conflict of interest statement:* none declared.
